# Mucinous breast carcinoma presenting as Paget's disease of the nipple in a man: A case report

**DOI:** 10.1186/1746-1596-3-42

**Published:** 2008-10-24

**Authors:** Dimitrios Peschos, Elena Tsanou, Pavlos Dallas, Konstantinos Charalabopoulos, Christos Kanaris, Anna Batistatou

**Affiliations:** 1 Dept. of Forensic Science, Medical Faculty, University of Ioannina, Ioannina, Greece; 2 Dept. of Physiology, Clinical Unit, Medical Faculty, University of Ioannina, Ioannina, Greece; 3 Dept. of Pathology, Medical Faculty, University of Ioannina, Ioannina, Greece; 4 Cytology Laboratory, K Frontzou 7, Ioannina, Greece; 5 Gynecologist, C. Tricoupi 6, Ioannina, Greece

## Abstract

**Introduction:**

Male breast cancer is rare compared to its female counterpart representing less than 1% of cancer in men. Moreover, mucinous carcinoma of the male breast is an extremely rare histological subtype of malignancy. Paget's disease of the nipple is rarely observed in males.

**Case report:**

Herein, we describe a unique case of an 86 years old man with mucinous breast cancer presenting as Paget's disease of the nipple. According to the immunohistochemical evaluation the neoplastic cells were positive for estrogen (ER) and progesterone receptors (PR).

**Conclusion:**

To our best knowledge this is the first case of mucinous male breast cancer presenting as Paget's disease of the nipple.

## Background

The existing epidemiological data confirms the infrequency of male breast cancer (MBC) which represents 1% of all breast cancers [1,2] Overall, the epidemiology of MBC presents similarities with the epidemiology of female breast cancer (FBC). Major genetic factors associated with an increased risk of breast cancer for men include BRCA2 mutations, which are believed to account for the majority of inherited breast cancer in men [3], Klinefelter syndrome, and a family history. Suspected genetic factors include AR gene mutations, CYP17 polymorphism, Cowden syndrome, and CHEK2 [3]. Epidemiologic risk factors for MBC include disorders relating to hormonal imbalances, such as obesity, testicular disorders (e.g., cryptorchidism, mumps orchitis, and orchiectomy), and radiation exposure. Suspected epidemiologic risk factors include prostate cancer, prostate cancer treatment, gynecomastia, occupational exposures (e.g., electromagnetic fields, polycyclic aromatic hydrocarbons, and high temperatures), dietary factors (e.g., meat intake and fruit and vegetable consumption), and alcohol intake [3].

Paget's disease presents as an eczematous change of the nipple and areola [4]. Specifically, it is characterized by a red, oozing, crusted lesion which is often unresponsive to topical steroid and antibiotics. There is almost always present an underlying breast carcinoma. The prevalence of an associated cancer ranges from 67–100% with most studies reporting the presence of a concurrent malignancy in over 90% of patients [5]. Most commonly, this is an infiltrating ductal carcinoma but occasionally a ductal carcinoma in situ (DCIS) may be present [4]. Overall, this type of cancer is rare, comprising 3% to 5% of all mammary malignancies [6].

To our best knowledge, about 30 cases of mucinous MBC and about 50 cases of Paget's disease of the male breast have been until now reported in the English literature [7-9]. Furthermore, the presentation of the mucinous breast carcinoma as Paget's disease of the male breast is considered to be unique.

## Case presentation

**Figure 1:**
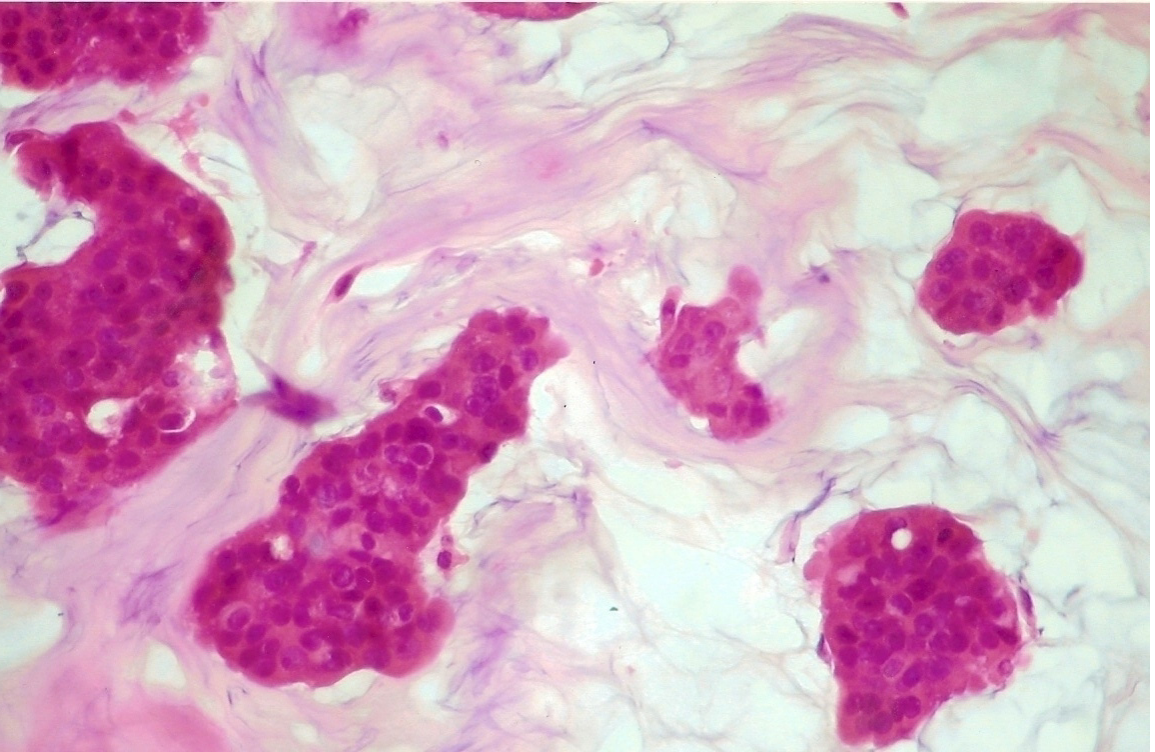
Histological evaluation revealed the presence of mucinous carcinoma (H + E, × 400).

**Figure 2:**
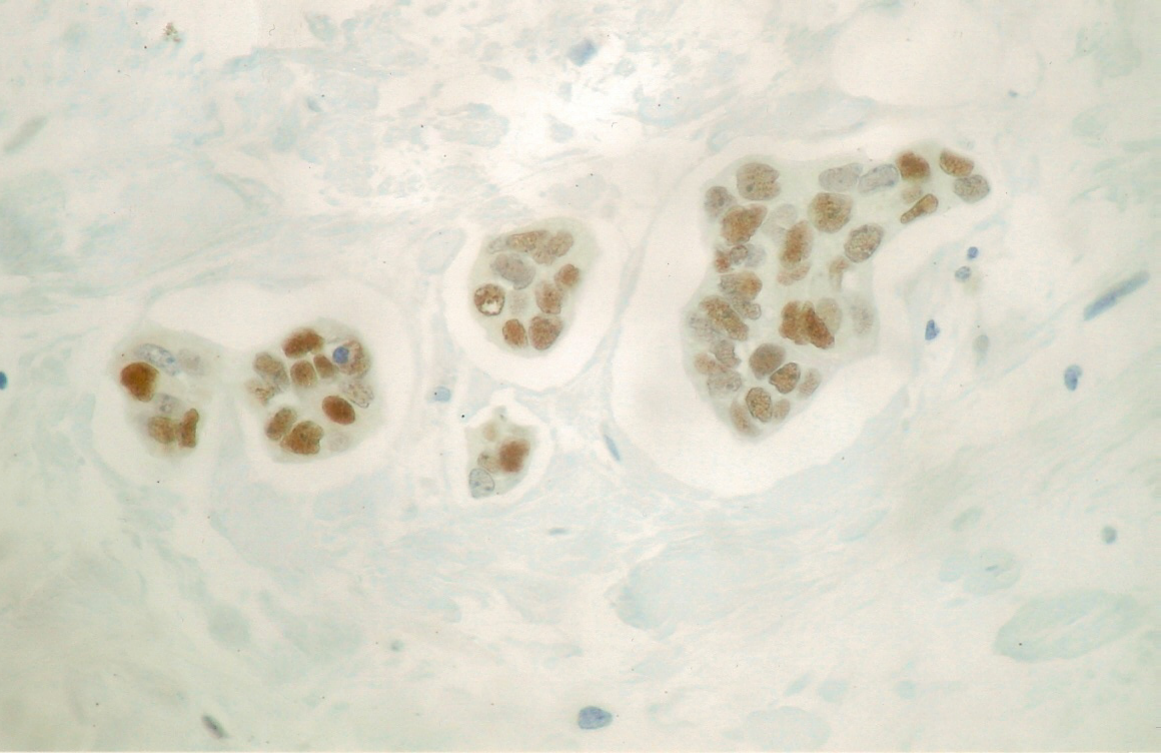
The neoplastic cell expressed ER&#945 receptors (DAB &#215 400)

An 86-year-old man with no medical history presented with a persistent erythema, oozing and scaling of the left nipple. The lesion was initially characterized as mammary Paget's disease, based on clinical signs. Additionally, there was no history of predisposing factors to breast lesions such as drug use (i.g. prostate cancer treatment), gynecomastia or hormonal imbalances, such as obesity and/or testicular disorders. Physical examination revealed a mass localized in the subareolar area. Fine needle aspiration biopsy was performed on the mass and malignancy was revealed on histopathological examination. The diagnosis was made after the histological examination of a breast gland segment (14 × 11 × 6 cm in size) covered by a spindle-shaped skin (13 × 9 cm in size) bearing the nipple. The mass was well circumscribed 5 × 7 × 6 cm in size of soft and friable consistency with mucoid texture. The result from the histological examination revealed mucinous carcinoma of the breast gland (grade II) (Figure 1). The diagnosis was based on the presence of large amounts of extracellular mucin, in the background and detached epithelial tumor ceels in trabecular and micro papillary formations. Fibrous stroma was sparse. Immunohistochemical evaluation revealed that the neoplastic cells were positive for estrogen (ER) (Figure 2) and progesterone receptors (PR). Therapeutically, after the modified radical mastectomy chemotherapy and tamoxifen was administered.

## Discussion

MBC constitutes less than 1% of all breast cancer cases [1,2]. Mucinous or colloid breast cancer is considered to be a rare subtype of breast cancer (invasive ductal breast cancer) and probably slightly more uncommon in men than in women [10]. Special types such as Paget's disease of the nipple and mucinous breast cancer in a man are extremely rare. To our best knowledge this is the first case reported of mucinous male breast cancer presenting as Paget's disease of the nipple.

We must emphasize that Paget's disease must be differentiated from other skin disorders, such as eczema, and it is usually the presence of an underlying lump that indicates the invasive nature of the lesion. In addition, melanoma and adenoma of the nipple may rarely mimic Paget's disease. The correct diagnosis requires histopathologic interpretation, as clinical signs may be similar.

According to the immunohistochemical analysis the cancer cells were estrogen and progesterone receptor positive. Studies have shown that MBC is more likely to be of high grade at presentation time, affecting mostly elderly males, with retained expression of ER and PR [2,10-13]. In our case reported herein, the patient was 86 years old. In specific, the majority of cancers arising in the male breast are ER positive although this finding does not correlate with a better prognosis, as it occurs in women [14,15] Clinical responses to hormonal therapy have been observed in the ER+ patients but not in one ER – patient [16]. In women, ER expression is usually a marker of differentiation and indicates that the cancer still remains under hormonal influence. This characteristic would also imply that the tumor should be less aggressive and more responsive to hormone therapy. In men, however, ER-positive tumors are associated with higher stage disease [15]. Postmenopausal women have been found to have tumors that are more likely to be hormone receptor expressers as well and follow a more indolent course [17]. One possibility is that hormone receptor-positive cancers are a consequence of aberrant steroid receptor up-regulation in the estrogen-starved postmenopausal setting. The tumors over expressing the steroid receptor may have constitutive activation of downstream targets. The fact that most cancers arising in male breasts are ER positive is likely due to the lack of circulating estrogen in the male system, much like in postmenopausal women [2]. Apart from steroid receptors other immunohistochemical differences between male and female breast cancer must also be considered. For instance, cases of MBC are less likely to overexpress p53 and Erb-B2, which are associated with survival and cell proliferative activity than the female counterparts [2].

It appears that MBC has a more aggressive clinical behavior than FBC with a worse outcome when compared stage for stage [18,19]. Some studies referred to worse prognosis in men mainly due to anatomic factors (i.e., paucity of breast tissue and close tumor proximity to skin and nipple, facilitating dermal lymphatic spread and early regional and distant metastasis) and delayed diagnosis [13,18,20]. Usually, in MBC symptoms are often delayed and the lag time between first symptoms and surgery in some cases is twice as long as in women [21]. In the case reported herein, the mammary Paget-like lesion of the skin bearing the nipple was the main symptom. On the other hand, numerous studies have shown that breast carcinoma in males is not biologically more aggressive than in females and the prognosis of patients with breast cancer is the same in male and female patients when disease-specific survival rate, tumour size and axillary involvement are compared [22-24].

Modified radical mastectomy, combined with sentinel-node biopsy by experienced teams, is the standard treatment in case of MBC. Criteria for adjuvant systemic treatment are identical for men and women, although hormonal therapy (tamoxifen) has a more prominent place in the adjuvant setting because of the high percentage of positive hormone receptors in men [25].

Owing to its infrequent occurrence, knowledge of the etiology, pathology, immunophenotype and behavior of MBC lags behind that regarding FBC. Numerous studies have confirmed distinct differences in the genetic basis of MBC and FBC [26,27]. The poorer prognosis of the male breast carcinoma might be related to ineffective therapies which do not consider these differences in the biological profile of the male tumor. Prognostic and predictive tissutal markers, detected by immunocytochemical methods and useful for therapeutic programming in the female breast cancer have a different significance in the male breast cancer and stress the need for different therapeutic strategies specific for male breast cancer.

## Conclusion

More research is needed on male breast cancer as it becomes more apparent that it is a different disease than its female counterpart. This recognition will provide better-focused treatment strategies and improved survival, and will perhaps even provide us with a better understanding of breast carcinogenesis both in men and women.

## List of abbreviations

MBC: male breast cancer; FBC: female breast cancer; DCIS: ductal carcinoma in situ; ER: estrogen receptor; PR: profesterone receptor.

## Consent

Written informed consent was obtained from the patient for publication of this case report and accompanying images. A copy of the written consent is available for review by the Editor in Chief of this journal.

## Competing interests

The authors declare that they have no competing interests.

## Authors' contributions

DP conceived the study and participated in the design and writing of the report. ET and AB made the final diagnosis and helped to draft the manuscript. PV carried out the immunoassay; KC made the critical appraisal. KC was the attending physician.
